# Evaluating Cognitive and Behavioral Outcomes in Conjunction with the Secure Base Effect for Dogs in Shelter and Foster Environments

**DOI:** 10.3390/ani9110932

**Published:** 2019-11-07

**Authors:** Lauren E. Thielke, Monique A. R. Udell

**Affiliations:** Animal & Rangeland Sciences, Oregon State University, Corvallis, OR 97331, USA; Monique.Udell@oregonstate.edu

**Keywords:** attachment behavior, attachment style, canine cognition, shelter dog behavior, foster dog behavior

## Abstract

**Simple Summary:**

Attachment relationships formed early in life have important implications for long-term social and behavioral outcomes for humans. Although previous research has established that dogs and humans form attachment relationships that can be categorized into attachment styles, the implications of these relationships have not been well-studied. This is a particularly important area of study for dogs living in foster homes and animal shelters, as it is currently unknown if and how attachment relationships formed in these environments correspond with behavioral and cognitive outcomes. In this study, we examined attachment styles in relation to cognitive measures and surveys of canine behavior and personality completed by caretakers of dogs in foster and shelter environments. Foster dogs with secure attachments displayed higher levels of persistence and performance on cognitive tasks compared to foster dogs with insecure attachments. On a survey given to foster and shelter volunteers, we found that securely attached dogs were rated as less neurotic than insecurely attached dogs for both foster and shelter groups. These results indicate that attachment relationships are associated with other important behavioral and cognitive traits of foster and shelter dogs, suggesting that the potential for causal associations should be explored further.

**Abstract:**

Although it is widely accepted that dogs and humans form attachment relationships, characterizing attachment styles in dogs has only recently received attention in the literature. Previous research has shown that pet dogs display patterns of behavior in an attachment test that can be classified into secure and insecure attachment styles, much like human children and their caretakers. However, we currently know relatively little about the role of attachment styles in relation to canine well-being. This question may be of particular interest for the 3.9 million dogs that enter animal shelters in the United States alone each year, as this transition marks the dissolution of prior bonds and the establishment of new attachment relationships. Herein, results are presented from analyses of volunteer-reported canine personality and behavior measures, as well as performance on two cognitive tasks as they relate to attachment styles developed within shelter and foster environments. Results from the Canine Behavioral Assessment and Research Questionnaire (C-BARQ) indicated that foster dogs were scored as having significantly higher levels of attachment and attention-seeking behaviors when compared with shelter dogs. In both environments, dogs categorized as securely attached to a shelter or foster volunteer had lower neuroticism scores. Secure attachment in foster homes was also associated with improved persistence and performance on a point following task. These results provide support for the idea that attachment styles formed with temporary caregivers is associated with other behavioral and personality measures, and therefore may have implications for behavior and welfare in dogs living in foster homes and animal shelters.

## 1. Introduction

Attachment relationships are close social bonds that often form between caregivers and their dependents. Previous research has shown that attachment styles can be classified as secure or insecure [1]. Secure attachments are characterized by an ability to seek comfort from a caregiver when exposed to stress-inducing stimuli and then engage in play or exploratory behavior, which is known as the secure base effect. Although individuals with insecure attachments will display a variety of patterns of behavior in a stressful situation, all of these patterns feature an inability to successfully receive comfort from a caregiver, resulting in either persistent distress or a lack of interaction with a caregiver [1]. Studies of attachment relationships in human children have indicated that attachment styles are an important component of behavioral and cognitive development. For example, insecure disorganized attachments have been found to be predictive of aggression in children both at the time of testing and years later [2,3]. Secure attachment to a caregiver as a toddler has been associated with better performance on assessments of executive functioning and higher-order cognitive processes such as impulse control and working memory in early childhood [4]. For children in institutionalized settings, placement in foster care (especially before 24 months of age) has been associated with higher rates of secure attachment formation to future caregivers [5] as well as lower rates of psychiatric disorders [6]. While most behavioral attachment testing is done with human infants and young children, attachment relationships can form across the human lifespan (often measured with surveys post-adolescence) and continue to act as an important predictor of other behavioral and cognitive outcomes [7,8].

Recent research has shown that domestic dogs form attachments bonds to their human owners that are similar (in form and function) to parent–offspring attachment in children, and that a dogs’ behavior in an attachment test can be used to categorize dog–human bonds into the same traditional attachment styles at roughly equivalent rates as found in human literature [9,10,11]. However, to date, only a few studies have examined the relationship between dog–human attachment and other measures of canine welfare, behavior, and cognition. In one such study, dogs spent significantly more time manipulating a toy containing a food reward in the presence of their owners than in the presence of an unfamiliar person, suggesting that attachment (and more specifically the secure base effect) may have important implications for persistence on cognitive tasks [12]. Another study demonstrated that therapy dogs with secure attachment to their handlers, spent a greater proportion of time focused on the therapy participant during a mock therapy session than did insecurely attached dogs [10].

Understanding how dog-human attachment relationships correspond with other measures of behavior and well-being may be of particular interest within shelter and foster populations, as the transition into shelter or foster marks the dissolution of prior bonds and presents the potential for establishment of new attachment relationships. Understanding the relationship between attachment and other behaviors in dogs could provide information valuable to management, care, and placement of these animals. Such transition periods may also present the greatest opportunity for fostering the development of new secure attachments. However, first, it is necessary to empirically evaluate attachment relationships between foster dogs, shelter dogs, and their respective caregivers, and to determine what implications, if any, these attachment relationships have on performance on cognitive tasks and behavioral measures. This study was designed to address some of these current knowledge gaps by (1) testing and categorizing attachment relationships between shelter and foster dogs and their caretakers and (2) determining if different attachment styles corresponded with different outcomes on a variety of behavioral, personality, or cognitive measures comprised of volunteer-completed the Canine Behavioral Assessment and Research Questionnaire (C-BARQ) and Monash Canine Personality Questionnaire-Revised (MCPQ-R) surveys as well as a spatial discrimination task and a pointing task. Object choice tasks, where a pointing gesture is used to indicate the location at which a food reward will be provided, have become an established method of measuring social cognition in canine populations [13,14,15]. Previous research has shown that pet dogs perform more successfully on a pointing task compared to stray shelter dogs, but that shelter dogs are also able to learn from previous trials and demonstrate improved performance after additional training trials [15]. We expected that foster dogs might show intermediate performance levels somewhere between what has been found in shelter and pet dogs, and that dogs with secure attachments would outperform those with insecure attachments. Based on findings in the human literature, we expected that dogs with a secure attachment to a shelter or foster volunteer would show fewer behavioral problems on the C-BARQ, score higher on extraversion and motivation, and lower on neuroticism on the MCPQ-R, demonstrate greater persistence on the spatial discrimination task, and perform better on a test of social cognition compared to insecurely attached dogs.

## 2. Materials and Methods

### 2.1. Subjects and Participants

Twenty-one foster dogs and 31 shelter dogs participated in this study. One dog was dropped from analyses of cognitive measures because cognitive tests could not be attempted due to the shelter closing before cognitive tasks could be completed. One foster dog was blind and therefore was dropped from pointing analyses, but successfully completed the location-based spatial discrimination task and was included in analysis for that test. All procedures with animals were approved by Oregon State University’s (OSU) Institutional Animal Care and Use Committee (Animal Care and Use Protocol #4837).

Foster participants included 20 foster parent volunteers. Shelter participants included 20 shelter volunteers at Willamette Humane Society in Salem, OR who interacted with dogs regularly as part of their volunteer duties. Each participant provided consent to participate in the study, covered under Institutional Review Board Protocol #7818. All surveys were anonymous and tracked via a participant number assigned by the researchers.

Prior studies have suggested that adult dogs living in a shelter can begin to show signs of attachment to humans after only three, 10-min interaction periods [13] and show signs of reduced stress (lower cortisol levels) after only 135 min of added human interaction implemented over 3 consecutive days [16]. Improved performance on socio-cognitive tests has been reported after 30 min of targeted social interaction with dogs in the shelter [17]. Therefore, we predicted that attachment effects would be present with as little as three separate 10-min interaction sessions, and thus served as our minimal criterion for interaction history between participating dyads.

### 2.2. Secure Base Test

All dogs participated in a secure base test (SBT) with their caretakers (shelter volunteers or foster volunteers). In all cases, testing was conducted in a mostly barren, unfamiliar room (e.g., adoption counseling room/behavioral evaluation room) at a site in close proximity to where the animal was residing. Each testing room contained a chair with a circle, 1-m in radius, taped on the floor around the chair and three dog toys. The SBT consists of three two-minute phases. In baseline, caretakers could freely interact with the dog when it entered the circle with two paws or half a body length (including playing, petting, talking, etc.) but were to remain neutral when the dog was not in the circle. In the alone phase, the caretaker left the room and the dog was alone. In the final phase, the caretaker returned and instructions were identical to baseline.

#### Video Analysis of SBT

Two independent coders reviewed the return phase videos for each dog’s SBT and categorized dogs’ attachment styles based on patterns of behavior seen in the return phase. Inter-rater reliability was assessed based on the percentage of agreement after this initial round of coding. After the two coders reviewed each video independently, they watched any videos for which they disagreed on attachment style categorization together and reached an agreement. If the coders could not agree on an attachment style for a particular dog, the dog would be categorized as “Unclassifiable” and removed from analysis. A description of all attachment style classifications can be found in Table 1. Independent agreement between coders for attachment style was 72%, and a consensus was reached for all dogs when coders reviewed videos together.

### 2.3. Spatial Discrimination Task

This test was conducted by an unfamiliar experimenter and handler (the dogs had not encountered either person prior to the day of the testing session). Dogs participated in training trials in which one bowl was placed on the floor in either a positive (food available) or negative (food unavailable) location [18,19]. Bowls were spaced 2 m apart, and a handler held the dog on a leash 3 m away from each bowl, consistent with prior research [20]. The experimenter stood in the middle of the two bowls, 1 m away from either side. For a diagram of the testing layout, please see [20]. The bowls were 30 × 20 cm wide and 12 cm deep. In half of the testing sessions, the right side of the room was designed as the positive location and in half of the testing sessions the container on the left side was designated as the positive location. No pre-baiting of bowls with food occurred, except on the first trial, which allowed dogs to see that a food reward was available in the positive bowl. In all subsequent trials, food was dropped into the container at the “positive location” only after the positive location was chosen. The container was removed after the dog ate the food reward that had been placed in it. No food was placed in the negative container when it was chosen. The container was removed after the dog approached and sniffed the container. For each trial, the experimenter faced the dog, established eye contact with the dog and said the dog’s name and “Look!” before placing the container at the designated location (i.e., only one container was used and was only present at the positive or negative location for each trial). Containers were presented in a fixed, semi-random order PPNPNN (P = positive, N = negative) [20]. The handler released the dog when the bowl was placed on the ground. If the dog did not move, the handler was allowed to encourage the dog by saying “Go!” or “Okay!”. The experimenter looked straight ahead without making eye contact with the dog once the dog was released by the handler.

For each trial, dogs were given 35 s to make a choice. If they did not approach either container with their snout within 10 cm of the container in 35 s, the trial was scored as “no choice”. Discrimination criteria was reached when, for the preceding five positive trials and preceding five negative trials, the longest latency to reach the positive location was shorter than any of the latencies to reach the negative location. We measured persistence at this task, defined as the total number of trials in which a container was chosen, to evaluate test performance.

### 2.4. Pointing Task

Testing Layout: An experimenter stood between two metal paint canisters 19 cm in height and 16 cm in diameter. Containers were spaced 1 m apart, and the experimenter stood directly in the middle of the two containers, 0.5 m from either side. Dogs were held on leash by a handler 2.5 m away from the experimenter and equidistant from the containers. For a diagram of the testing layout, please see [15].

Pretraining: Before the pointing test dogs participated in a test of motivation in which the experimenter placed food on each of two testing containers over two consecutive trials. If the dog ate the food on each of these trials it moved on to the pointing task.

Pointing task: Neither container was pre-baited. The experimenter called the dog’s name and then pointed to the target container using a momentary distal point, in which the experimenter extended her ipsilateral arm and hand in a traditional point in the direction of the correct container for two seconds before returning her arm to a neutral position at her side [15]. At full extension the tip of the experimenter’s finger was 50 cm from the top of the container. Dogs were given 30 s to make a choice (i.e., for their snout to come within 10 cm of a container). If they chose correctly food was dropped on the container for the dog to consume, and then the dog was taken back to the starting point by the handler. If the dog went to the other container, no food was provided and the dog was taken back to the starting point by the handler. If the dog did not choose within 30 s, no food was provided, the trial was counted as “no choice” and the dog was taken back to the starting point by the handler for the next trial.

### 2.5. Survey Measures

Foster and shelter volunteers were asked to complete a shortened version of the Canine Behavioral Assessment and Research Questionnaire (C-BARQ) that has previously been validated for use in animal shelters [21] and the Monash Canine Personality Questionnaire-Revised (MCPQ-R) [22] while dogs participated in cognitive tasks with the experimenters in a separate location. The Monash Canine Personality Questionnaire (MCPQ-R) has previously been validated and used for assessments of canine personality on five traits: motivation, training focus, amicability, extraversion, and neuroticism [22].

### 2.6. Statistical Methods

All statistics were two-tailed with an alpha level of 0.05 unless otherwise noted. Pointing score data and measures of persistence on both cognitive tasks were non-normally distributed. Normality was evaluated using a Shapiro–Wilk test, all *p* < 0.05. A Shapiro–Wilk test was used to assess normality for subcategories on the MCPQ-R and the C-BARQ. For the MCPQ-R, extraversion, motivation, training focus, and amicability were normally distributed, all *p* > 0.05. The MCPQ-R subcategory of neuroticism was non-normal, *p* < 0.05. Additionally, *t*-tests with a Tukey–Kramer adjustment for pairwise comparisons were used for all data, as most categories were normally distributed, and *t*-tests are robust to violations of normality. We also assessed differences between groups (shelter vs. foster) and differences between attachment styles (secure vs. insecure) on each MCPQ-R trait using *t*-tests with a Tukey–Kramer adjustment for pairwise comparisons. All C-BARQ trait data of interest were non-normally distributed (Shapiro–Wilk, *p* < 0.05), therefore non-parametric Mann–Whitney U tests were used for analyses of C-BARQ data.

## 3. Results

### 3.1. Cognitive Measures

**Figure 1:**
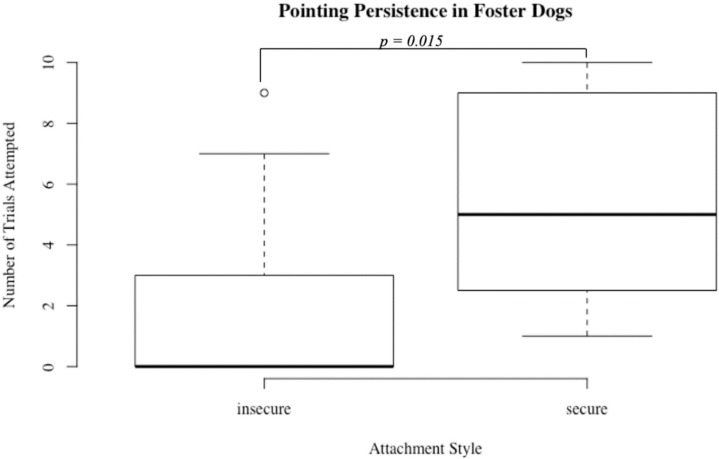
Persistence at a pointing task in foster dogs by attachment style. Persistence was measured as the total number of trials on which a choice was made, and dogs with secure attachments (*N* = 11) to volunteers persisted for significantly more trials than dogs with insecure attachments (*N* = 9) to volunteers, *p* < 0.05.

**Figure 2:**
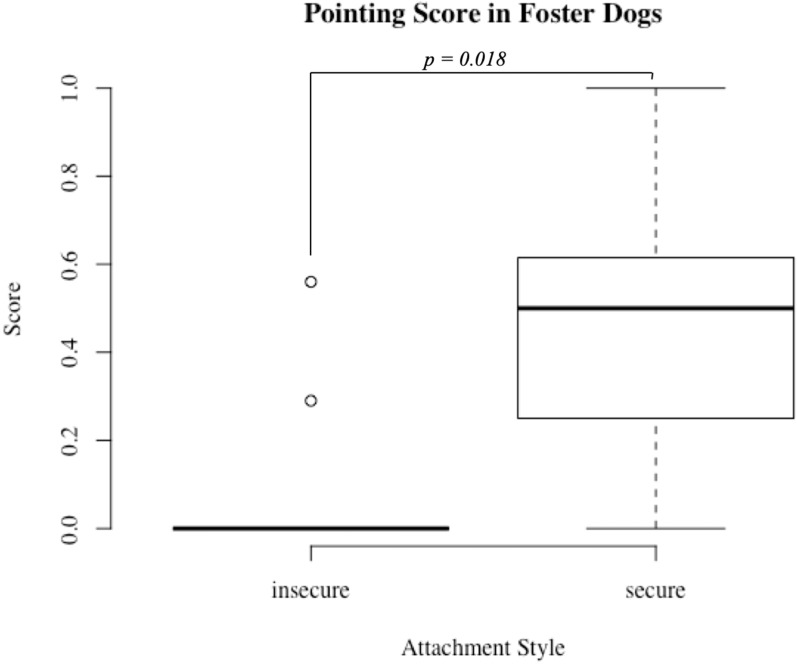
Pointing score in insecure and secure foster dogs. Foster dogs with secure attachments (*N* = 11) to volunteers scored significantly higher (more correct choices out of total number of choices made) than foster dogs with insecure attachments (*N* = 9) to volunteers, *p* < 0.05.

Within each group, we evaluated persistence on two cognitive tasks in conjunction with attachment style. For foster dogs, dogs with secure attachments to a foster volunteer completed significantly more trials (out of ten total) on the pointing task compared to dogs with insecure attachments indicating greater task persistence (secure median number completed = 5 insecure = 0, U = 17.5, *p* = 0.02; Figure 1). Additionally, foster dogs with secure attachments had significantly higher scores on the pointing task compared to insecure foster dogs (secure median percent correct = 50%, insecure = 0%, U = 20, *p* = 0.02; Figure 2). There was also a trend with respect to secure dogs persisting for more trials at a spatial discrimination task (secure median number completed = 7, insecure = 2, U = 30, *p* = 0.09). For the shelter group, dogs with secure and insecure attachments to shelter volunteers did not differ with respect to persistence or performance on either the spatial discrimination task or the pointing task (all *p* > 0.05).

### 3.2. Survey Measures

#### 3.2.1. MCPQ-R

**Figure 3:**
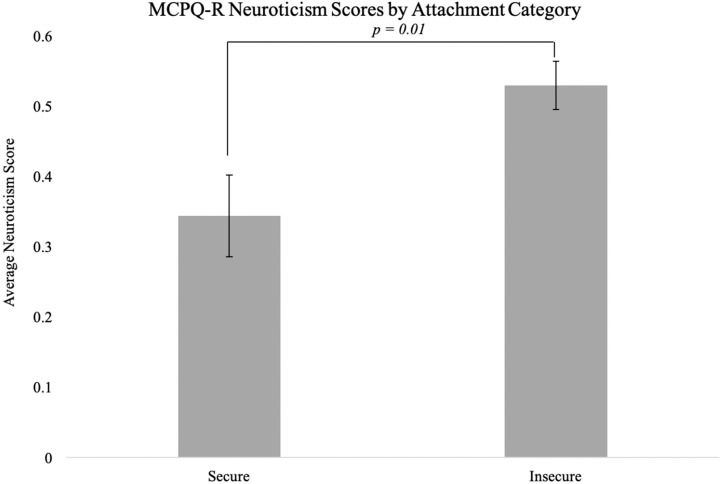
Mean neuroticism score for insecure (*N* = 28) and secure (*N* = 24) dogs based on attachment style. Error bars indicate the standard error of the mean.

Across both shelter and foster dogs, insecure dogs scored significantly higher on the neuroticism scale than secure dogs (mean insecure dogs = 0.53, secure dogs= 0.34, *t* (39) = 2.74, *p* = 0.01; Figure 3). A trend was also found with respect to motivation (mean insecure dogs = 0.56, secure dogs = 0.67, *t* (39) = –1.88, *p* = 0.07). No significant differences were found with respect to training focus, extraversion, or amicability (all *p* > 0.05).

In order to investigate whether there was a relationship between dogs’ environment and volunteer-reported behavior on MCPQ-R scales, we conducted pairwise comparisons for each subscale with respect to group. There was a trend of shelter dogs scoring higher on the extraversion subscale (mean shelter dogs = 0.71, foster dogs = 0.59, *t* (39) = −1.76, *p* = 0.09). Shelter dogs received an average score of 0.71 on extraversion, and foster dogs were given an average score of 0.59 on the extraversion subscale. No significant differences were found between groups for scores on motivation, training focus, amicability, or neuroticism (all *p* > 0.05).

#### 3.2.2. C-BARQ

**Figure 4:**
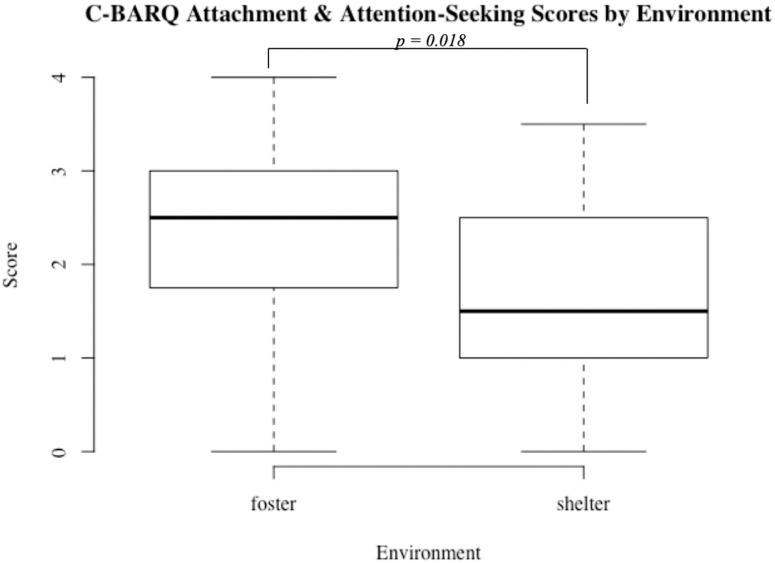
Attachment and attention seeking scores in foster (*N* = 20) and shelter (*N* = 20) dogs.

For C-BARQ data, we investigated whether group (foster vs. shelter) or attachment style (secure vs. insecure) predicted volunteer reports of separation-related problems, attachment and attention seeking, or stranger-related fear. No significant differences were found between securely and insecurely attached dogs for these measures (all *p* > 0.05). However, a significant difference between the shelter and foster populations was found for the attachment and attention seeking measure, with foster dogs scoring significantly higher on the attachment scale on average (median shelter dogs = 1.5, foster dogs = 2.5, U = 299.50, *p* = 0.02; Figure 4). There was also a significant difference between populations with respect to volunteer-reported separation-related behavior problems (median shelter dogs = 0.85, foster dogs = 1.67, U = 286, *p* = 0.047). No significant population differences were found with respect to stranger-related fear, *p* > 0.05.

## 4. Discussion

The results of the current study demonstrate that shelter and foster dogs can form impactful attachment bonds with volunteers, although attachment strength and the influence of attachment security may be greater within the foster environment, at least on some measures. We also found that attachment security did correspond with superior performance on at least some cognitive tasks (persistence and gesture responsiveness in fostered dogs), and lower neuroticism scores (in both shelter and foster dogs) as reported by a volunteer caretaker. While it should be noted that causality, or the direction of the relationship between these measures and attachment style cannot be determined from these results, such outcomes are consistent with prior findings in the human literature [4,23,24].

While more research is needed on dog–human attachment relationships in general, it may be especially important to understand attachment relationships in foster and shelter populations, as dogs in these settings may be more likely to face welfare challenges, changes in attachment figures and in some cases may experience reduced opportunity for social interaction [25]. Furthermore, dogs in shelter environments may be exposed to a variety of volunteers, staff, and potential adopters each day. Given that shelter dogs have been shown to form attachment bonds with new people after only three ten-minute interaction sessions [17], it is important to understand how attachment bonds affect behavior and welfare in shelter settings. Links between attachment security in shelter and foster environments and cognitive and behavioral traits may help shed light on ways that dog–human bonds can be used to improve welfare or predict outcomes during this critical time of transition.

It is also important to note that random assignment of dogs into shelter and foster settings was not possible for the current study, future studies may aim to address this variable. However, there may also be practical challenges to truly random assignment. For example, foster dogs are often selected because of medical problems, behavior problems, or to alleviate stress experienced in the shelter environment. Such factors may also influence attachment relationships or serve as more powerful predictors of certain behavioral or cognitive outcomes. In fact, some general differences between dogs in foster care vs. shelter housing were identified, independent of attachment style, including volunteer-reported attachment and attention-seeking behavior and separation-related problems. Foster dogs with secure attachments displayed better overall cognitive performance and persistence on a cognitive task, but shelter dogs did not show this pattern, thus secure bonds formed in a foster home may allow for more beneficial outcomes. Although relatively few studies have investigated outcomes of foster care, one study found that foster dogs had lower rates of return than shelter dogs [26] and lower cortisol:creatinine ratios after even a short stay in a foster home [27]. In conjunction with our findings, the current research on foster programs provides evidence that foster opportunities are likely beneficial in terms of welfare and behavioral outcomes.

As a whole, this study provides evidence that secure attachment styles to volunteer caregivers are associated with improved performance and persistence on a cognitive task for foster dogs, and lower levels of neuroticism in both shelter and foster dogs. These findings are in line with research in human children in foster care showing that secure attachment relationships are associated with more positive mental health outcomes [6]. Future research could examine what factors help facilitate the formation of secure bonds for dogs in foster settings. Additional research focusing on adoption outcomes of dogs in foster and shelter settings is also important for gaining a better understanding of how to facilitate secure attachment formation in dogs’ adoptive homes.

## 5. Conclusions

This study provides evidence that attachment relationships in shelter and foster dogs are related to important behavioral and cognitive outcomes. These results are more prevalent in foster dogs, which may be related to stronger attachments among foster dog-foster volunteer dyads compared to shelter dog-shelter volunteer dyads. These results are in line with findings from research with human children and outcomes related to caregiver attachment styles. Future research should continue to explore the implications of attachment styles for dogs in a variety of settings.

## Figures and Tables

**Table 1:** Holistic coding attachment style definitions (adapted from [11]).

Attachment Style	Definition
Secure	Little or no resistance to contact or interaction. Greeting behavior is active, open, and positive. Seeks proximity and is comforted upon reunion, returning to exploration or play.
Insecure ambivalent	Shows exaggerated proximity-seeking and clinging behavior, but may struggle if held by owner. Mixed persistent distress with efforts to maintain physical contact and/or physically intrusive behavior directed toward the owner. (Dogs who the judges agreed seemed essentially secure but with ambivalent tendencies, were included in the secure group).
Insecure avoidant	May show little/no distress on departure. Little/no visible response to return, ignores/turns away but may not resist interaction altogether (e.g., rests or stands without bodily contact, out of reach or at a distance).
Insecure disorganized	Evidence of strong approach avoidance conflict or fear on reunion, for example, circling owner, hiding from sight, rapidly dashing away on reunion, “aimless” wandering around the room. May show stereotypies on return (e.g., freezing or compulsive grooming). Lack of coherent strategy shown by contradictory behavior. “Dissociation” may be observed, that is, staring into space without apparent cause; still or frozen posture for at least 20 s (in the nonresting, nonsleeping dog).
Unclassifiable	Classifiers were unable to reach consensus on group placement for dogs from this classification category. Unclassifiable dogs were excluded from further analysis on dog attachment.
